# Edible Safety Evaluation of *Cinnamomum camphora* Seed Kernel Oil: Sub-Chronic Toxicity and Teratogenicity Assessments

**DOI:** 10.3390/foods14122116

**Published:** 2025-06-17

**Authors:** Xianghui Yan, Ting Peng, Zheling Zeng, Pengbo Wang, Yifang Gao, Xuefang Wen, Jiaheng Xia, Deming Gong, Ping Yu

**Affiliations:** 1 State Key Laboratory of Food Science and Resources, Nanchang University, Nanchang 330047, China; xianghui_y@163.com (X.Y.); ncu_pt@163.com (T.P.); dgong01@gmail.com (D.G.); 2 College of Food Science and Technology, Nanchang University, Nanchang 330031, China; 3 School of Chemistry and Chemical Engineering, Nanchang University, Nanchang 330031, China; k24038882@kcl.ac.uk (P.W.); gyf10107329@163.com (Y.G.); 4 Department of Nutritional Sciences, School of Life Course and Population Sciences, Faculty of Life Sciences and Medicine, King’s College London, London SE1 9NH, UK; 5 Institute of Applied Chemistry, Jiangxi Academy of Sciences, Nanchang University, Nanchang 330031, China; wen_xuefang@live.cn; 6 Key Laboratory of Monitoring and Assessment on Novel Food Raw Materials, State Administration for Market Regulation, Chengdu Institute of Food Inspection, Chengdu 611130, China; xia_jheng@163.com; 7 New Zealand Institute of Natural Medicine Research, 8 Ha Crescent, Auckland 2104, New Zealand

**Keywords:** no-observed-adverse-effect level, long-term oral toxicity, teratogenicity, safety assessment, medium chain triglycerides

## Abstract

Medium chain triglycerides (MCTs) are regarded as an important ingredient for functional foods and nutraceuticals. *Cinnamomum camphora* seed kernel oil (CCSKO) contains more than 95% medium chain fatty acids (MCFAs), which is a significantly higher level than palm kernel oil (62%) and coconut oil (55%). However, the safety assessment of CCSKO, as the only natural MCT oil rich in capric acid and lauric acid found so far in the world, has not been fully verified. The study aimed to investigate the 90-day sub-chronic oral toxicity and teratogenicity of CCSKO. In the sub-chronic oral toxicity study, no clinically significant adverse events occurred in male or female Sprague–Dawley (SD) rats with CCSKO daily administration for 13 weeks. Moreover, there were no dose–response relationships between CCSKO and body-weight gain, food intake and food utilization in male or female SD rats. No significant differences (*p* > 0.05) were found in the hematological properties or organ weights between the male and female SD rats. In the teratogenicity test, no toxicological signs were observed in either Wister pregnant rats or fetuses. The no-observed-adverse-effect level of CCSKO was determined to be more than 4 mL/kg body weight. These results suggested that CCSKO may be an excellent edible oil with high oral safety.

## 1. Introduction

The digestion, absorption, transport and metabolism of medium-chain triglycerides (MCTs) are different from those of long-chain triglycerides (LCTs) in vivo [1,2,3]. MCTs are easily completely hydrolyzed to medium-chain fatty acids (MCFAs) and glycerol by lipase in the gastrointestinal tract, and absorbed into the blood by small intestinal epithelial cells. MCFAs in the cytoplasm can directly enter the mitochondria and be oxidized to produce energy, or converted to ketones and oxidized to produce energy without binding with carnitine [1,2,3]. As a result, most of the absorbed MCT is used to supply energy in vivo and not converted to fat and stored in the body [4,5,6,7]. In terms of health benefits, MCTs can significantly alleviate glucose and lipid metabolic disorders [8,9,10,11,12,13,14]. Moreover, MCTs have the potential to exert ketogenic effects, thereby controlling epileptic seizures, preventing neurodegeneration in Alzheimer’s disease, and enhancing memory in older individuals [15,16,17,18].

MCTs were listed as “Generally Recognized as Safe” (GRAS) substances by the Food and Drug Administration (FDA), USA in 1994, and as a safe raw food raw material or emulsifier by the National Health Commission of P. R. China in 2013. They have been widely used as drug delivery media [4,6] and food ingredients for special dietary purposes [15,19,20]. However, there are very limited resources of natural oils rich in MCFAs, especially MCTs, in the world. Currently, the commercial MCTs are mainly produced by hydrolysis, fractionation and esterification from palm kernel oil (PKO) and coconut oil (CO), which contain about 62% (*w*/*w*) and 55% (*w*/*w*) of MCFAs, respectively [21,22].

*Cinnamomum camphora* seeds are woody oilseeds rich in MCT oil and protein, and *Cinnamomum camphora* seed kernel (CCSK) contains 46.29–62.08% (*w*/*w*) of MCT oil and 12.33–20.07% (*w*/*w*) of protein [23]. The CCSK oil (CCSKO) contains 0.41–2.57% (*w*/*w*) of caprylic acid (C8:0), 51.49–61.23% (*w*/*w*) of capric acid (C10:0), and 35.83–40.08% (*w*/*w*) of lauric acid (C12:0). The MCFA content of CCSKO is more than 95% (*w*/*w*), which is 1.6 times and 1.7 times that of PKO and CO, respectively [24]. Moreover, CCSKO contains 97% (*w*/*w*) MCTs, and can significantly alleviate glucose and lipid metabolic disorders in high-fat-diet-induced obese rats [25,26], which is consistent with the physiological effects of MCTs. These reports indicate that CCSKO is not only a natural MCT oil with several physiological properties in vivo, but also an ideal raw material to produce medium- and long-chain triglycerides (MLCTs) and structured triglycerides (STGs) for special diet purposes, with broad development prospects [27]. Notably, CCSKO was recorded as being used as a cooking oil in Anfu County, Jiangxi Province, China [24]. It was also verified that CCSKO had no acute oral toxicity in mice and did not cause genotoxicity [mammalian erythrocyte micronucleus test, Ames’s test, and in vitro mammalian cell TK gene mutation test; and the median lethal dose (LD_50_) value of CCSKO was higher than 21.5 g/kg body weight (BW)] [24]. However, to the best of our knowledge, the information about long-term oral toxicity and teratogenicity test of CCSKO is limited.

In this study, the effects of long-term feeding with different doses of CCSKO on the growth and physiology of SD rats were first observed through repeated-dose oral toxicity testing of rats. The repeated exposure dose–response relationship, target organ toxicity and reversibility, and no observed adverse effect level (NOAEL) of CCSKO were studied to evaluate the chronic oral toxicity of CCSKO. Then, the teratogenicity of CCSKO in Wistar pregnant mice and fetal mice was studied through teratogenicity testing. Finally, based on the results of the acute toxicity and genotoxicity assessment of CCSKO completed before, the edible safety of CCSKO was determined through comprehensive analysis, which provided the scientific basis for developing CCSKO as a new food material.

## 2. Materials and Methods

### 2.1. Chemical Reagents

Paraformaldehyde, xylene, ethanol, hematoxylin, eosin, paraffin, Bouin’s fixative solution, alizarin red, potassium hydroxide, acetic acid and glycerin were purchased from Shanghai Aladdin Biochemical Technology Co., Ltd. (Shanghai, China). Reagents for hematology analysis, blood biochemical analysis, blood coagulation analysis and urine analysis were purchased from Thermo-Fisher Scientific Inc. (Waltham, MA, USA). All other solvents and reagents used were of analytical grade.

### 2.2. Instruments and Equipment

The instruments and equipment used were as follows: rat surgical kits (Kent Scientific Co., Ltd., Torrington, CT, USA), electronic scales (Thermo-Fisher Scientific Inc., Waltham, MA, USA), Zeiss Primo Star microscope (Carl Zeiss AG Co., Ltd., Baden-Württemberg, Germany), JY-A-XPC ophthalmoscope (Shanghai Shenguang Co., Ltd., Shanghai, China), BC-5300Vet automated hematology analyzer (Mindray, Shenzhen, China), Urit-610 coagulation analyzer (URIT Co., Ltd., Guilin, China), Urit-910c fully automated electrolyte analyzer (URIT Co. Ltd., Guilin, China), Urit-500B urine analyzer (URIT Co., Ltd., Guilin, China), BeneHeart R12a electrocardiogram scanner (Shenzhen Mindray Bio-Medical Electronics Co., Ltd., Shenzhen, China), Sorvall ST 16R Centrifuge (Thermo-Fisher Scientific Inc., Waltham, MA, USA), microtome BK-2268 (Olabo Scientific Instrument Co., Ltd., Jinan, China), electronic digital display calipers (0–150 mm) (Shanghai Measuring Tools and Cutting Tools Factory Co., Ltd., Shanghai, China), CX41 biological microscope (Olympus Corporation of Japan, Tokyo, Japan), and Motic SMZ168-BL stereo microscope (McDior Industrial Group Co., Ltd., Xiamen, China).

### 2.3. Test Substance

The test substance CCSKO was produced from *Cinnamomum camphora* seeds by using the aqueous enzyme extraction (AEE) method as described in our previous study [24].

### 2.4. Sub-Chronic Toxicity Evaluation of CCSKO

#### 2.4.1. Animals and Housing Environment

One hundred and twenty specific pathogen-free (SPF) grade SD rats (half male and half female) with BW between 65 g and 85 g were provided by Hunan Shrek Jingda Laboratory Animal Co., Ltd. (Changsha, China) (License number: SCXK (Xiang) 2016–0002). The rats were housed in an animal room of Jiangxi Provincial Center for Disease Control and Prevention, China (Qualification certificate: No. SYXK (Gan) 2012–0003) at a temperature of 22.1–24.7 °C, relative humidity of 50–60% and a 12 h light/dark cycle. The basic animal feed was provided by Hunan Shrek Jingda Laboratory Animal Co., Ltd. (Changsha, China) (License number: SCXK (Xiang) 2016–0002).

#### 2.4.2. Study Design

The animal study was performed at the Jiangxi Provincial Center for Disease Control and Prevention (Nanchang, China) (Qualification certificate: No. SYXK (Gan) 2012–0003), in accordance with the “90-day Oral Toxicity Test in Chinese National Standard for food safety” (GB 15193.13-2015) [28]. The animal experiment was approved by Jiangxi Provincial Center for Disease Control and Prevention, China. All the animals used were cared for in accordance with the Guide for the Care and Use of Laboratory Animals, 8th edition (Committee for the Update of the Guide for the Care and Use of Laboratory Animals, 2011). All procedures were approved by the Experimental Animal Ethics Committee, Nanchang University, China (Approval Code: No. 20211215-0115).

#### 2.4.3. Animal Feeding

After 3 days of acclimation, the rats were randomly divided into 6 groups (*n* = 20, 10 males and 10 females), including 2 satellite groups (treated for 6 weeks) and 4 experimental groups (treated for 13 weeks). For the two satellite groups, rats were fed with 4 mL/kg BW (high-dose group, HDG-S) and 0 mL/kg BW (control group, CG-S) of CCSKO, respectively. For the four experimental groups, rats were fed with 4 mL/kg BW (high-dose group, HDG-E), 2 mL/kg BW (medium-dose group, MDG-E), 1 mL/kg BW (low-dose group, LDG-E) and 0 mL/kg BW (control group, CG-E) of CCSKO, respectively. HDG-S and CG-S indicate that CCSKO was replaced with 0% and 100% of soybean oil, respectively. HDG-E, MDG-E, LDG-E and CG-E indicate that CCSKO was replaced with 0%, 50%, 75% and 100% of soybean oil, respectively. Each rat was housed in a single cage and free to eat and drink.

#### 2.4.4. General Clinical Observation of SD Rats

The mortality, morbidity and clinical observations of rats were recorded once a day during the dosing period. Ophthalmological examinations of rats were carried out during both pre-dosing and end-of-dosing periods. BW and food consumption of rats were measured weekly.

#### 2.4.5. Hematology, Clinical Biochemistry and Urinalysis of SD Rats

The clinical pathology evaluations (hematology and clinical biochemistry) were performed during both pre-dosing and end-of-dosing periods, urinalysis was performed at the end of the dosing period only (week 6 for satellite groups and week 13 for experimental groups, respectively). Whole blood samples were obtained via intracardiac injection under anesthesia (isoflurane) after fasting overnight. The parameters in the hematological examination are listed as follows: white blood cell count and differential leucocyte count (neutrophils, lymphocytes, intermediate cells), red blood cell count, hemoglobin concentration, hematocrit, platelet count, prothrombin time (PT) and activated partial thromboplastin time (APTT).

Serum biochemistry evaluation was performed using an automatic chemistry analyzer (Urit-8060, URIT Co., Ltd., Guilin, China). The following clinical biochemistry parameters were analyzed on the serum obtained by centrifugation of the aforementioned blood samples: alanine aminotransferase (ALT), aspartate aminotransferase (AST), glutamyl transpeptidase (GTT), alkaline phosphatase (ALP), blood urea nitrogen (BUN), creatinine (CRE), total cholesterol (TC), triglycerides (TG), glucose (GLU), total protein (TP), albumin (ALB), chlorine (Cl), potassium (K) and sodium (Na).

Urine samples were collected from the rats at week 6 for satellite groups and week 13 for experimental groups. The urinary protein content, specific gravity, pH value, glucose level, and occult blood of urine were evaluated. Color and turbidity were examined visually.

#### 2.4.6. Gross Necropsy, Organ Weight and Histopathological Observation of SD Rats

At the end of the dosing period, each rat was anesthetized with isoflurane, and none of the rats regained consciousness before euthanasia. Then, a complete gross necropsy was performed. Histopathological evaluation was conducted in brain, lung, liver, spleen, stomach, intestine, testis, ovary, epididymis, uterus, heart and kidneys. All samples were weighted prior to fixation in 10% formalin. Tissues were embedded in paraffin and 4 μm-thick sections were obtained and stained with hematoxylin–eosin. The slides were scanned, and representative pictures were extracted for illustration purposes.

### 2.5. Teratogenicity Evaluation of CCSKO

#### 2.5.1. Animals and Housing Environment

Forty-eight male (360–500 g) and ninety-six female (250–350 g) specific pathogen-free (SPF) grade Wistar rats were provided by Hubei Experimental Animal Research Center (Wuhan, China) (License number: SCXK (E) 2020–0018; qualification certificate: No. 42000600055092). The rats were housed in an animal room of Hubei Provincial Center for Disease Control and Prevention (Wuhan, China) (Qualification certificate: No. SYXK (E) 2022–0065) at a temperature of 20–26 °C, relative humidity of 40–70% and a 12 h light/dark cycle. Basic animal feed was provided by Wuhan Wanqianjiaxing Biological Technology Co., Ltd. (Wuhan, China) (License number: SCXK (E) 2021–0011).

#### 2.5.2. Study Design

The animal study was performed at the Hubei Provincial Center for Disease Control and Prevention (Wuhan, China) (Qualification certificate: No. SYXK (E) 2022–0065), in accordance with the “Teratogenicity Test in Chinese National Standard for food safety” (GB 15193.14-2015) [29]. The animal experiment was approved by Hubei Provincial Center for Disease Control and Prevention (Wuhan, China). All the animals used were cared for in accordance with the Guide for the Care and Use of Laboratory Animals, 8th edition (Committee for the Update of the Guide for the Care and Use of Laboratory Animals, 2011). All procedures were approved by the Experimental Animal Ethics Committee, Nanchang University, China (Approval Code: No. 20211215-0116).

#### 2.5.3. Animal Feeding

Female and male rats were housed in the same cage for mating in a ratio of 2:1. Then, the pregnant rats were found the next day and randomly assigned to 4 groups with 16–17 rats in each group. The day of positive vaginal smear or plug was considered as day 0 of gestation. The pregnant rats were fed 4 mL/kg BW (high-dose group, HDG-T; *n* = 16), 2 mL/kg BW (medium-dose group, MDG-T; *n* = 17), 1 mL/kg BW (low-dose group, LDG-T; *n* = 16) and 0 mL/kg BW (control group, CG-T; *n* = 16) of CCSKO, from day 6 to day 15. HDG-T, MDG-T, LDG-T and CG-T indicate that CCSKO was replaced by 0%, 50%, 75% and 100% of soybean oil, respectively. Each rat was housed in a single cage and free to eat and drink. On day 20, all rats were euthanized, and the maternal conception and fetal development were examined by laparotomy.

#### 2.5.4. General Clinical Observation of Pregnant Rats

The mortality, morbidity and clinical observations including skin, coat, eyes, mucosa, respiration, neurobehavior, and limb movement of pregnant rats were recorded once a day during the experiment. BW and food consumption of pregnant rats were measured at day 0, day 6, day 9, day 12, day 15 and day 20.

#### 2.5.5. Examination of Fetal Rats

At the end of the dosing period, the uterus was quickly removed and weighed. The number of corpora luteum, stillbirths, embryo/fetal absorptions, live and dead fetuses, implantation and sex ratios were recorded. The rates of implantation, live/dead fetuses and embryo/fetal absorptions were calculated by the following Equations (1)–(4):

(1) $$Implantation\;rate\left(\%\right)=\frac{Number\;of\;implantations}{Number\;of\;corpus\;luteum}\times 100\%$$

(2) $$Live\;fetus\;rate\left(\%\right)=\frac{Number\;of\;live\;births}{Number\;of\;fetal\;rats}\times 100\%$$

(3) $$Still\;fetus\;rate\left(\%\right)=\frac{Number\;of\;stillbirths}{Number\;of\;fetal\;rats}\times 100\%\;$$

(4) $$Embryo/fetal\;absorption\;rate\left(\%\right)=\frac{Number\;of\;absorbed\;fetuses}{Number\;of\;implantation}\times 100\%$$

Then, the uterus was dissected to observe the status of the fetal rats. The general appearance of teratogenicity, including encephalocele, open brain, small head, small ears, small eyes, cleft lip, short limbs, and multiple toes of fetal rats, were examined. The body length and weight of fetal rats were also recorded. After examination and measurement, half of the live fetal rats were fixed in Bouin’s solution for 2 weeks, then the visceral abnormalities were examined. The other half of the live fetal rats were immersed in ethanol (95%, *v*/*v*) for 3 weeks, cleared in potassium hydroxide (2%, *w*/*w*) for 3 days, and then stained with Alizarin red solution for 2 days to evaluate skeletal abnormalities. The rates of total and single teratogenesis in each group were calculated by the following Equations (5)–(6):

(5) $$Total\;teratogenesis\;rate\;of\;each\;group\left(\%\right)\;=\frac{Number\;of\;live\;litters\;with\;malformations}{Number\;of\;litters}\times 100\%$$

(6) $$Single\;teratogenesis\;rate\;of\;each\;group\left(\%\right)=\frac{Number\;of\;live\;litters\;with\;a\;certain\;malformation}{Number\;of\;litters}\times 100\%$$

### 2.6. Statistical Analysis

The data generated by the experiment were collated and counted using Microsoft Excel 2021 software (Microsoft Corporation, Redmond, WA, USA). Results are expressed as the mean ± standard deviation (SD). Males and females were evaluated separately according to dose and compared with their respective control groups. Data were analyzed using one-way analysis of variance (ANOVA), followed by a post-hoc Tukey’s test with SPSS 26.0 software (SPSS, Inc., Chicago, IL, USA). Differences with a *p*-value of less than 0.05 were considered statistically significant.

## 3. Results and Discussion

### 3.1. The Chronic Toxicity of CCSKO in SD Rats

According to the guidelines GB 15193.13-2015 [28] and GB 15194.14-2015 [29], for administrating oily liquids, the maximum gavage concentration should be no more than 4 mL/kg BW, and the gavage volume in each group should be the same. Additionally, the interval between the groups of decreasing metering should be 2 to 4 times. Therefore, the dosages in this research were set as 4 mL/kg BW, 2 mL/kg BW, 1 mL/kg BW, and 0 mL/kg BW, and all CCSKO was dissolved in soybean oil.

#### 3.1.1. General Clinical Observations of SD Rats Fed with CCSKO

The chronic oral toxicity test is the most common method used in non-clinical safety assessment for novel natural products [30]. The test aims to determine the toxicity of subjects via oral administration repeatedly in 90 days, and determine the dose–reaction relationship, least-observed-adverse-effect level (LOAEL), no-observed-adverse-effect level (NOAEL) and oral safety of subjects. Therefore, in this study, a 90-day oral-toxicity test was used to assess the potential toxicity of CCSKO and identify vulnerable target organs induced by CCSKO.

The changes in general clinical behaviors are important markers of the toxic effects of CCSKO [31]. The results showed that no animal mortality occurred during the experiment in either satellite groups (HDG-S, CG-S) or experimental groups (HDG-E, MDG-E, LDG-E and CG-E). Before and after the experiment, no conjunctival secretion, congestion, edema, corneal, iris, lens opacity and retinal hemorrhage or other abnormal manifestations were found in either satellite groups or experimental groups by ophthalmic examinations. In addition, no treatment-related changes in locomotor activity or other behaviors were observed during the feeding period. A previous study reported that long-term MCT consumption was not detrimental to maintaining psychological health, including boosting memory and improving social behaviors in rats [32], which is consistent with our study. These results preliminarily suggested that CCSKO was safe for oral consumption since no clinically significant adverse events occurred when it was administrated daily to SD rats for up to 13 weeks.

#### 3.1.2. BW, Food Consumption and Efficiency of SD Rats Fed with CCSKO

As shown in Figure S1A, the BW of HDG-E, MDG-E, LDG-E and CG-E gradually increased during the experiment. Compared to rats before administration, the BW gains of the male HDG-E, MDG-E, LDG-E and CG-E rats at week 13 were 596.81%, 641.11%, 624.97% and 611.94%, respectively; the BW gains of the female HDG-E, MDG-E, LDG-E and CG-E rats at week 13 were 329.43%, 343.20%, 328.04% and 332.86%, respectively. There were no significant differences (*p* > 0.05) in the weekly and final BW in either the male or female HDG-E, MDG-E, LDG-E and CG-E rats. Similarly, no significant changes (*p* > 0.05) were found in the weekly and final food intake (Figure S1B), BW gain (Figure S1C) or food utilization rates (Figure S1D) between the male or female HDG-E, MDG-E, LDG-E and CG-E rats. No significant changes (*p* > 0.05) were found in the total BW gain (Figure S2A), food intake (Figure S2B) and food utilization rate (Figure S2C) between the male or female HDG-E, MDG-E, LDG-E and CG-E rats. Similar results were also found in satellite groups (HDG-S, CG-S) (Figures S3 and S4), indicating that there were no dose–response relationships between CCSKO and BW, BW gain, food intake and food utilization of the SD rats. In addition, the water consumption levels were similar among all groups in both genders.

#### 3.1.3. Hematological Properties, Biochemistry, Electrolyte and Urine Parameters of SD Rats Fed with CCSKO

**Figure 1:**
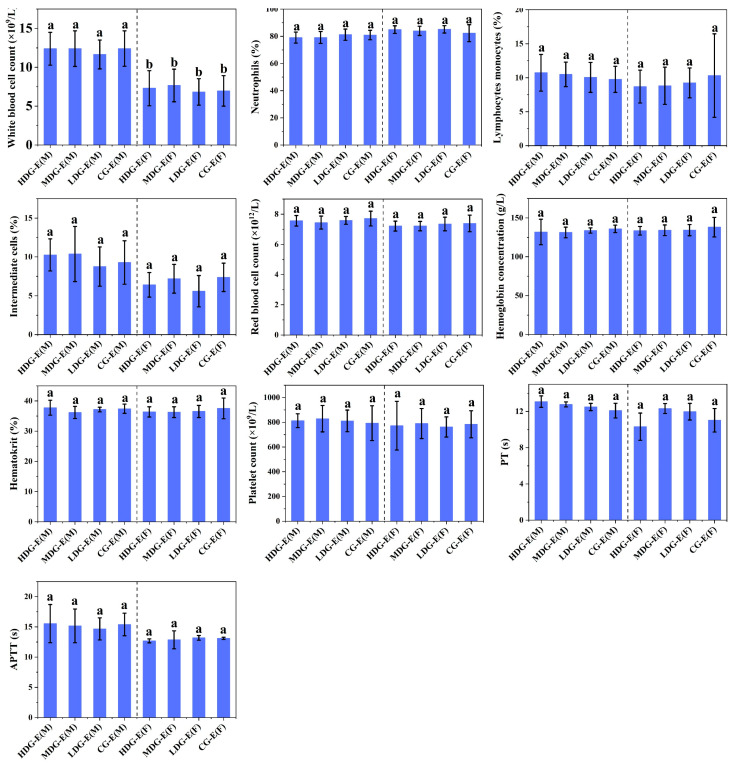
Effect of CCSKO on hematology, including blood routine (white blood cell count, neutrophils, lymphocytes, intermediate cells), leukocyte differential count (red blood cell count, hemoglobin concentration, hematocrit, platelet count) and blood coagulation analysis (PT, APTT) of SD rats in experiment groups (13 weeks) of sub-chronic toxicity. HDG-E(M), MDG-E(M), LDG-E(M) and CG-E(M) indicate high-dose group (4 mL/kg BW), medium-dose group (2 mL/kg BW), low-dose group (1 mL/kg BW) and control group (0 mL/kg BW) of male rats in experiment groups, respectively; HDG-E(F), MDG-E(F), LDG-E(F), and CG-E(F) indicate high-dose group (4 mL/kg BW), medium-dose group (2 mL/kg BW), low-dose group (1 mL/kg BW), and control group (0 mL/kg BW) of female rats in experiment groups, respectively. PT, prothrombin time; APTT, activated partial thromboplastin time. Data are expressed as mean ± SD (*n* = 10). Values with different letters (a, b) indicate significant differences (*p* < 0.05). No significant differences (*p* > 0.05) were found between control and treatment groups in either sex.

No significant differences (*p* > 0.05) were observed in the hematological properties (Figure 1) between the male or female rats in experiment groups (HDG-E, MDG-E, LDG-E and CG-E) during the 13-week test period, including white blood cell count, neutrophils, lymphocytes monocytes, eosinophils and basophils, which can be considered as no dose–response relationship between CCSKO and the hematological properties of SD rats. Meanwhile, there were no significant differences (*p* > 0.05) in red blood cell count, hemoglobin concentration, hematocrit, platelet count, prothrombin time (PT), or activated partial thromboplastin time (APTT) among all groups between male and female rats. Similarly, no significant differences (*p* > 0.05) were found in the hematological properties between the male or female rats in the satellite groups (HDG-S, CG-S) during the 6-week test period (Figure S5).

**Figure 2:**
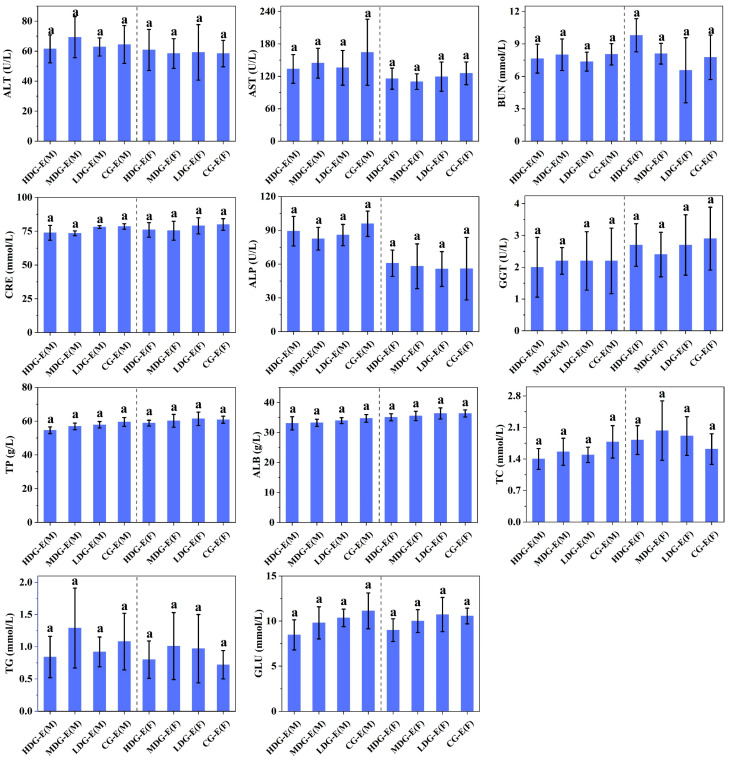
Effect of CCSKO on clinical biochemistry parameters of SD rats in experiment groups (13 weeks) of sub-chronic toxicity. HDG-E(M), MDG-E(M), LDG-E(M) and CG-E(M) indicate high-dose group (4 mL/kg BW), medium-dose group (2 mL/kg BW), low-dose group (1 mL/kg BW) and control group (0 mL/kg BW) of male rats in experiment groups, respectively; HDG-E(F), MDG-E(F), LDG-E(F) and CG-E(F) indicate high-dose group (4 mL/kg BW), medium-dose group (2 mL/kg BW), low-dose group (1 mL/kg BW) and control group (0 mL/kg BW) of female rats in experiment groups, respectively. ALT, alanine aminotransferase; AST, aspartate aminotransferase; BUN, blood urea nitrogen; CRE, creatinine; ALP, alkaline phosphatase; GTT, glutamyl transpeptidase; TP, total protein; ALB, albumin; TC, total cholesterol; TG, triglycerides; GLU, glucose. Data are expressed as mean ± SD (*n* = 10). Values with same letter (a) indicate no significant differences (*p* > 0.05) were found between control and treatment groups.

**Figure 3:**
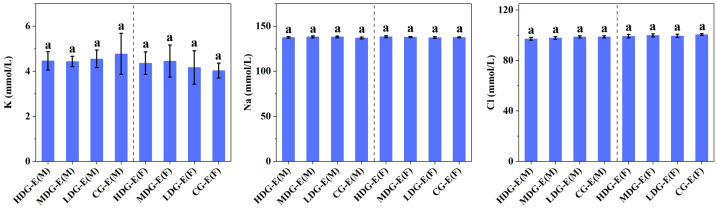
Effect of CCSKO on blood electrolytes (K, Na, Cl) of SD rats in experiment groups (13 weeks) of sub-chronic toxicity. HDG-E(M), MDG-E(M), LDG-E(M) and CG-E(M) indicate high-dose group (4 mL/kg BW), medium-dose group (2 mL/kg BW), low-dose group (1 mL/kg BW) and control group (0 mL/kg BW) of male rats in experiment groups, respectively; HDG-E(F), MDG-E(F), LDG-E(F) and CG-E(F) indicate high-dose group (4 mL/kg BW), medium-dose group (2 mL/kg BW), low-dose group (1 mL/kg BW) and control group (0 mL/kg BW) of female rats in experiment groups, respectively. Data are expressed as mean ± SD (*n* = 10). Values with same letter (a) indicate no significant differences (*p* > 0.05) were found between control and treatment groups.

**Figure 4:**
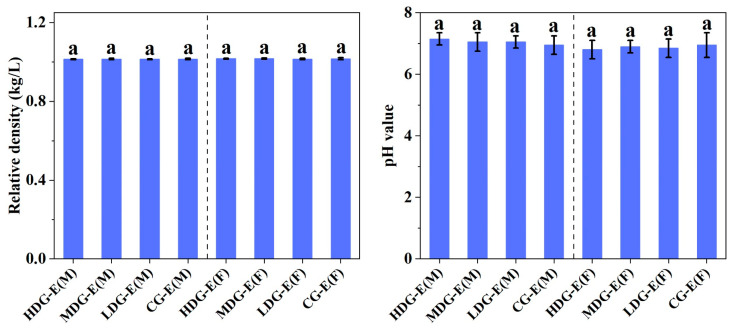
Effect of CCSKO on urinalysis (relative density, pH value) of SD rats in experiment groups (13 weeks) of sub-chronic toxicity. HDG-E(M), MDG-E(M), LDG-E(M) and CG-E(M) indicate high-dose group (4 mL/kg BW), medium-dose group (2 mL/kg BW), low-dose group (1 mL/kg BW) and control group (0 mL/kg BW) of male rats in experiment groups, respectively; HDG-E(F), MDG-E(F), LDG-E(F) and CG-E(F) indicate high-dose group (4 mL/kg BW), medium-dose group (2 mL/kg BW), low-dose group (1 mL/kg BW) and control group (0 mL/kg BW) of female rats in experiment groups, respectively. Data are expressed as mean ± SD (*n* = 10). Values with same letter (a) indicate no significant differences (*p* > 0.05) were found between control and treatment groups.

Similar to the hematological properties, no significant differences (*p* > 0.05) were observed in the blood biochemical parameters (Figure 2) and blood electrolytes (Figure 3) between male or female HDG-E, MDG-E, LDG-E and CG-E rats. Moreover, there were no significant differences (*p* > 0.05) in the relative density, pH value (Figure 4), urinary albumen positive, glucose positive or occult blood positive between male or female HDG-E, MDG-E, LDG-E and CG-E rats. Similar effects were also observed in the rats of the satellite groups (HDG-S and CG-S) (Figures S6–S8). In addition, the urine of all rats was observed to be clear and transparent, faint yellow to light yellow.

#### 3.1.4. Macroscopic Examinations, Organ Weights and Histopathological Observations of SD Rats Fed with CCSKO

**Figure 5:**
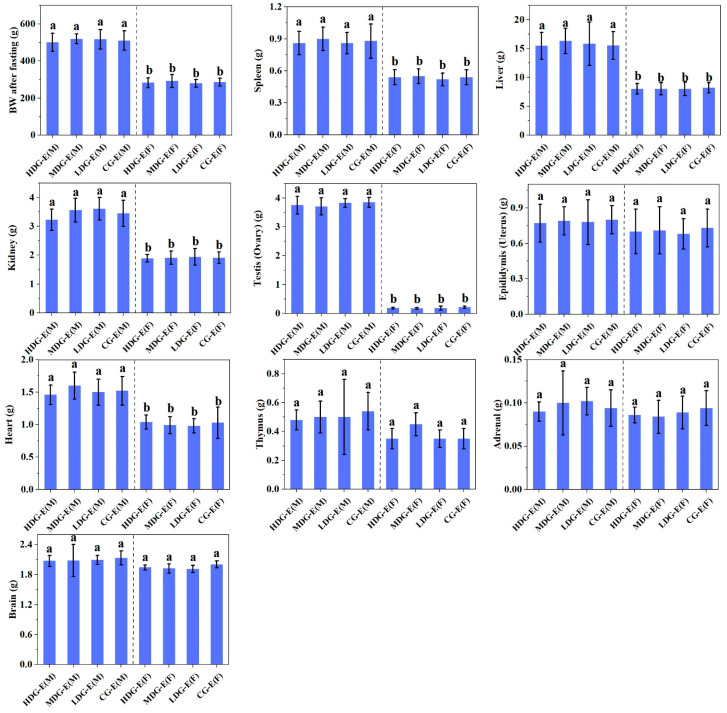
Effect of CCSKO on organ weights of SD rats in experiment groups (13 weeks) of sub-chronic toxicity. HDG-E(M), MDG-E(M), LDG-E(M) and CG-E(M) indicate high-dose group (4 mL/kg BW), medium-dose group (2 mL/kg BW), low-dose group (1 mL/kg BW) and control group (0 mL/kg BW) of male rats in experiment groups, respectively; HDG-E(F), MDG-E(F), LDG-E(F) and CG-E(F) indicate high-dose group (4 mL/kg BW), medium-dose group (2 mL/kg BW), low-dose group (1 mL/kg BW) and control group (0 mL/kg BW) of female rats in experiment groups, respectively. Data are expressed as mean ± SD (*n* = 10). Values with different letters (a, b) indicate significant differences (*p* < 0.05). No significant differences (*p* > 0.05) were found between control and treatment groups in either sex.

The specific composition of fatty acids and the structure of triglycerides have a key role in regulating visceral fat accumulation, thereby exerting an impact on the morphology and weight of organs [33]. As shown in Figure 5, there were no significant differences (*p* > 0.05) in the organ weights in male and female HDG-E, MDG-E, LDG-E and CG-E rats after 90-day repeated CCSKO administration, including BW after fasting, and the weights of spleen, liver, kidneys, testes (ovaries), epididymides (uterus), heart, thymus, adrenal gland and brain. Similarly, there were no significant differences (*p* > 0.05) in the organ weights between male or female HDG-S and CG-S rats (Figure S9).

As shown in Figures S10 and S11, no significant changes were observed in the macroscopic inspection of organs and tissues between male and female HDG-E and CG-E rats. The histological observations in the HDG and CG rats showed that there were no obvious abnormalities, including exudation, hyperplasia, edema, atrophy and other lesions in all the tissues and organs examined. The detailed description of the histological observations was as follows:

(A) Brain: The neurons in the cerebral cortex were arranged normally in layers, with no hemorrhage and edema, no atrophy in the cerebral cortex, and no degeneration or necrosis of neurons. The molecular layer of the cerebellar cortex, the Purkinje cell layer, and the granular layers were distributed normally, and there were no glial cells in the medulla hyperplasia. The basilar arteries in the hypothalamic–pontine section were normally distributed, the normal distribution of the transverse pontine bundle was observed below the basilar artery, with the normal distribution of the longitudinal pontine bundle and the medial lemniscus on the left and right sides.
(B) Lung: The bronchial wall and alveolar septum at all levels of the lung tissue were intact, and there was no pulmonary edema or hemorrhage, no necrosis of lung tissue cells, no inflammatory cell infiltration and no interstitial fibrosis.
(C) Liver: The hepatic lobules were intact; the hepatocytes were neatly arranged and the bile canaliculi and capillary bile ducts were normal.
(D) Spleen: The structure of the white pulp and red pulp of the spleen parenchyma was clear, and there was no abnormal proliferation or decrease in lymphocytes.
(E) Stomach: The gastric mucosa was intact, and there was no mucosal epithelial necrosis, shedding or hemorrhage.
(F) Intestine: The mucosal surface of the intestine was flat, with no villi, isolated lymph nodes in the lamina propria, or intestine hemorrhage, necrosis, ulceration and inflammatory cell infiltration.
(G) Testis and ovary: All levels of spermatogenic cells and mature sperm were observed in the testis, and there was no abnormality in the interstitium. The ovaries were well developed, with follicles and mature corpus luteum at all levels, with no ovarian cysts, no ovarian hemorrhage and no interstitial abnormalities.
(H) Epididymis and uterus: Many efferent tubules and epididymal ducts were observed in the epididymis; a large number of sperm cells and spermatocytes were seen in the official cavity, and there was no abnormality in the interstitium. The endometrium was normal, with no hyperplasia and inflammatory cell infiltration, no bleeding in the myometrium, and no abnormality in the outer membrane.
(I) Heart: There was no degeneration or necrosis of myocardial fibers, no inflammatory cell infiltration in the endocardium and epicardium, no hypertrophy and atrophy of myocardial cells and no interstitial vascular hemorrhage.
(J) Kidneys: The structure of the renal cortex and medulla was clear, and the distribution of nephrons was uniform. No abnormal pathological changes were observed in the epithelial cells of the glomerular capillary bundles, renal vesicles and renal tubules. No vascular dilation or inflammatory exudation was observed in renal interstitium, with no transitional epithelium of renal pelvis mucosa, no squamous metaplasia and no inflammatory cell infiltration.

Similarly, no significant changes were found in male and female HDG-S and CG-S rats (Figures S12 and S13). These results were consistent with Fu et al. [26], who found that CCSKO did not cause adverse effects on organ coefficients or organ morphology.

### 3.2. The Teratogenicity Evaluation of CCSKO in Wistar Rats

During the experiment, no treatment-related clinical signs of toxicity or mortality were observed in the pregnant rats fed with CCSKO at different doses (HDG-T, MDG-T, LDG-T and CG-T). Moreover, there were no significant differences (*p* > 0.05) in BW, BW gain (BW gain = maternal BW—BW on day 6) and net BW gain (net BW gain = maternal BW—uterine and fetal weight—BW on day 6) in the pregnant rats treated with CCSKO at any dose (Table 1). Compared with CG-T, there were no significant differences (*p* > 0.05) in the number of corpora luteum, implantation, live and dead fetuses, embryo/fetal absorptions, stillbirths, and sex ratios of pregnant rats in HDG-T, MDG-T, and LDG-T (Table 2). In addition, there were no adverse effects on uterine weight with fetus, fetal weight, fetal length and external malformation in pregnant rats exposed to different doses of CCSKO (HDG-T, MDG-T, LDG-T and CG-T) (Table 3). In terms of visceral examination, the main manifestation of visceral malformations in fetal rats was hydronephrosis. However, no significant differences (*p* > 0.05) were observed in the number of abnormal visceral organs and the rate of abnormal litters among the HDG-T, MDG-T, LDG-T, and CG-T groups (Table 4). On the other hand, the skeletal malformation of fetal rats was mainly manifested as the absence of the 5th and 6th sternum or ossification (Table 5). Skeletal abnormalities of fetal rats in all the groups were observed, which might have been the effect of the breeding environment. Nevertheless, there were no significant differences (*p* > 0.05) in the rate of skeletal malformations between the treated groups (HDG-T, MDG-T, LDG-T) and the negative control group (CG-T). Consequently, no adverse effects of CCSKO on the reproductive toxicity of pregnant rats or the development of fetal rats were observed. The NOAEL of CCSKO in the teratogenicity test was determined to be more than 4 mL/kg BW.

Based on the aforementioned results, the oral administration of CCSKO at doses up to 4 mL/kg BW neither resulted in mortalities, nor induced ophthalmological, clinical, BW, food consumption, or food efficiency changes. Furthermore, no adverse events were observed in clinical chemistry, hematology, coagulation, urinalysis, organ weights, or histopathology during the dosing phase in rats for either sex. Additionally, CCSKO demonstrated no adverse effects on the reproductive toxicity of pregnant rats or the development of fetal rats. Any observed changes in HDG rats compared with CG rats during the 90-day oral toxicity test and teratogenicity test were not dose-dependent. These changes were of low magnitude and consistent with historical control values, lacking toxicological significance. The results of this study demonstrated that CCSKO possessed similar toxicity effects to those of other currently marketed natural oils and supported the oral safety of MCT-rich oil [34]. These findings were consistent with a previous report by Matulka et al. [35], who determined the safety of MCTs when fed to beagles for 6 weeks at levels of 5%, 10% and 15% of MCT added to conventional feed. No signs of toxic effects were observed in any of the animals, and the animal survival rate was 100% at the end of the study. As reviewed by Traul et al. [36], caprylic-capric-MCT-rich oil has long been proven to have no toxic effects, as confirmed by a series of animal studies, such as a 3-week dietary toxicity study in chicks, 30-day oral gavage toxicity study in rats, 90-day parenteral toxicity study in rabbits, 3-month oral toxicity study in rats, and 3-month dietary toxicity study in rats. In addition, humans treated with parenteral MCTs have tolerated doses of 3.0–9.0 g/kg BW per day for several months without adverse effects [36]. Nevertheless, this is the first study to demonstrate the long-term oral safety of CCSKO, a natural capric-lauric-MCT-rich oil. Overall, the NOAEL of CCSKO in the chronic toxicity test and the teratogenicity evaluation were both greater than 4 mg/mL BW, and the human equivalent dose (HED) of CCSKO was calculated to be 0.65 mL/kg BW based on a simple practice guide by Nair et al. [37], which might provide a safety window for CCSKO consumption. Utilizing an adult with a BW of 60 kg and the CCSKO density of 0.93 g/mL [24] as a point of reference, the HED was calculated to be 39.00 mL or 36.27 g. Nevertheless, since the intragastric volume of the experimental animals was restricted in accordance with national guidelines, the NOAEL obtained from the experiment could not reach the true level of no visible harmful effects.

However, the long-term replacement of LCTs in the diet with MCTs may not be feasible due to the lower smoking point and higher bubble capacity of LCT [38]. On the other hand, LCT oils such as soybean oil and flaxseed oil, are rich in linoleic acid (C18:2) and linolenic acid (C18:3), which are essential for maintaining the normal physiological functions of the human body [39,40]. Hence, a possible solution is to develop CCSKO as an MCT ingredient to produce medium- and long-chain triglycerides (MLCT). There are several investigations that indicate that CCSKO can be synthesized as MLCTs to improve the physicochemical properties of physically blended MCT/LCT [41,42]. In addition, many studies have reported that MLCTs exhibited different metabolic characteristics and nutritional values [27], and were generally considered safe and edible [43,44]. Matulka et al. [45] found that the MLCTs did not show significant adverse effects, with the administration of MLCTs to rats at 3.5 g/kg BW per day for 6 weeks. Zhou et al. [46] evaluated the safety of a novel MLCT with 30% (*w*/*w*) MCFAs as a dietary fat in KM mice and SD rats at various doses (2.0–8.0 g/kg BW per day), and found no dose-related adverse effects in the rats administered with different doses of MLCTs. Further safety assessments in pregnant rats did not find any significant differences relative to the control at treatment doses up to 8.0 g MLCT/kg BW per day. All these studies indicated the safe use of MLCTs with a certain content of MCFAs in food products for improving human health. Therefore, the long-term consumption of MCFAs containing CCSKO is recommended. Our results suggest that CCSKO has great potential to become a valuable resource of edible MCT oil for use in diverse food products, including MLCTs and nutrient delivery systems.

## 4. Conclusions

The 90-day sub-chronic oral toxicity test and teratogenicity test of CCSKO at different doses (0, 1, 2 and 4 mL/kg BW) were evaluated in this study. The results showed that CCSKO did not induce mortalities or ophthalmological and clinical abnormalities in SD rats during the dosing period. Moreover, there were no adverse events in the clinical chemistry, hematology, coagulation, urinalysis, organ weights, or histopathology between the treated and negative control groups. Additionally, CCSKO demonstrated no adverse effects on the reproductive toxicity of pregnant rats or the development of fetal rats. These results indicated that CCSKO had no sub-chronic oral toxicity and teratogenicity and its NOAEL was higher than 4 mL/kg BW. In our previous study, CCSKO showed no acute oral toxicity, mutagenicity or genotoxicity. Therefore, CCSKO is a kind of natural and safe edible MCT oil, which has great health benefits for consumers and a huge potential market. Further studies on the toxicology, pharmacology and bioaccessibility of CCSKO in humans are needed for CCSKO to be applied to the production of medical products and foods for medical use.

## Figures and Tables

**Table 1:** Effects of CCSKO on BW of pregnant rats.

Groups	Number of Pregnant Rats	BW (g)							
		Day 0	Day 6	Day 9	Day 12	Day 15	Day 20	BW Gain	Net BW Gain
CG-T	16	282.4 ± 19.1	317.0 ± 18.2	326.1 ± 19.4	347.1 ± 19.9	367.2 ± 19.1	433.5 ± 32.2	116.5 ± 26.3	35.5 ± 15.8
LDG-T	16	282.4 ± 11.4	318.9 ± 13.0	329.4 ± 15.7	349.3 ± 14.5	368.9 ± 16.8	444.5 ± 26.5	125.5 ± 22.4	38.9 ± 22.0
MDG-T	17	281.6 ± 11.8	318.8 ± 15.4	329.4 ± 17.5	344.9 ± 18.8	369.7 ± 24.1	443.6 ± 29.4	124.8 ± 22.5	32.0 ± 16.3
HDG-T	16	281.5 ± 13.7	317.9 ± 14.1	328.9 ± 15.5	346.9 ± 15.5	367.5 ± 20.0	439.6 ± 28.2	121.7 ± 18.2	39.6 ± 12.2

CG-T, LDG-T, MDG-T and HDG-T indicate control group, low-dose group, medium-dose group and high-dose group in teratogenicity test, respectively. BW: body weight; BW gain = maternal BW—BW on day 6; net BW gain = maternal BW—uterine and fetal weight—BW on day 6. Data are expressed as mean ± SD.

**Table 2:** Effects of CCSKO on embryo formation in pregnant rats.

Groups	Number of Corpora Luteum	Number of Implantations	Number of Live Fetuses	Sex Ratio (Female/ Male)	Number of Stillbirths	Number of Absorbed Fetuses	Stillbirth Rate (%)	Absorbed Fetal Rate (%)
CG-T	301	219	212	106/106	0	7	0.00	3.20
LDG-T	314	244	232	100/132	0	12	0.00	4.92
MDG-T	339	257	252	120/132	0	5	0.00	1.95
HDG-T	306	230	220	103/117	0	10	0.00	4.35

CG-T, LDG-T, MDG-T and HDG-T indicate control group, low-dose group, medium-dose group and high-dose group in teratogenicity test, respectively.

**Table 3:** Effects of CCSKO on BW, body length, and appearance of abnormalities in fetal rats.

Groups	Number of Fetal Rats	Weight of the Uterus with the Fetus (g)	BW (g)	Body Length (mm)	Appearance Deformity					Number/Rate of Appearance Abnormalities (%)
					Encephalocele	Edema	No toe	Total	Deformity Rate (%)	
CG-T	212	81.0 ± 22.8	3.95 ± 0.25	62.59 ± 1.62	0	0	0	0	0.0	0/0.0
LDG-T	232	86.6 ± 12.7	3.75 ± 0.32	61.42 ± 1.71	0	0	0	0	0.0	0/0.0
MDG-T	252	92.8 ± 20.3	4.14 ± 0.65	62.18 ± 3.16	0	0	0	0	0.0	0/0.0
HDG-T	220	83.9 ± 13.3	3.94 ± 0.27	61.97 ± 1.36	0	0	0	0	0.0	0/0.0

CG-T, LDG-T, MDG-T and HDG-T indicate control group, low-dose group, medium-dose group and high-dose group in teratogenicity test, respectively. The weight of the uterus with the fetus, BW (body weight), and body length are expressed as mean ± SD.

**Table 4:** Effects of CCSKO on the viscera of fetal rats.

Groups	Number of Fetal Rats	Internal Organs Deformity and Abnormal Cases						Number/Rate of Viscera Abnormalities (%)
		Ventricular Abnormalities/Hypoplasia	Eyes Were Absent or Varied in Size	Cleft Palate	Hydronephrosis	Total	Deformity Rate (%)	
CG-T	101	0	0	0	1	1	1.0	1/6.3
LDG-T	113	0	0	0	0	0	0.0	0/0.0
MDG-T	122	0	0	0	3	3	2.5	2/11.8
HDG-T	105	0	0	0	5	5	4.8	4/25.0

CG-T, LDG-T, MDG-T and HDG-T indicate control group, low-dose group, medium-dose group and high-dose group in teratogenicity test, respectively.

**Table 5:** Effects of CCSKO on skeleton of fetal rats.

Groups	Number of Fetal Rats	Number of Skeletal Deformities and Abnormalities						Number/Rate of Skeleton Abnormalities (%)
		Interparietal Bone and Posterior Skull Were Missing	Sternum Was Absent and Incompletely Calcified	Rib Abnormalities (Multiple Ribs, Missing Ribs, Wavy Ribs)	Number of Spinal Bones Was Abnormal	Total	Deformity Rate (%)	
CG-T	111	0	6	0	0	6	5.4	4/25.0
LDG-T	119	0	6	0	0	6	5	3/18.8
MDG-T	130	0	8	0	0	8	6.2	4/23.5
HDG-T	115	0	5	0	0	5	4.3	5/31.3

CG-T, LDG-T, MDG-T and HDG-T indicate control group, low-dose group, medium-dose group and high-dose group in teratogenicity test, respectively.

## Data Availability

The original contributions presented in the study are included in the article/Supplementary Material, further inquiries can be directed to the corresponding authors.

## Supplementary Materials

The following supporting information can be downloaded at: https://www.mdpi.com/article/10.3390/foods14122116/s1, Figure S1: Effects of CCSKO on body weight (A), food intake (B), body weight gain (C) and food utilization rate (D) of SD rats in experiment groups (13 weeks) of sub-chronic toxicity; Figure S2: Effects of CCSKO on total body weight gain (A), total food intake (B) and total food utilization rate (C) of SD rats in experiment groups (13 weeks) of sub-chronic toxicity; Figure S3: Effects of CCSKO on body weight (A), food intake (B), body weight gain (C) and food utilization rate (D) of SD rats in satellite groups (6 weeks) of sub-chronic toxicity; Figure S4: Effects of CCSKO on total body weight gain (A), total food intake (B) and total food utilization rate (C) of SD rats in satellite groups (6 weeks) of sub-chronic toxicity; Figure S5: Effect of CCSKO on blood routine, leukocyte differential count and blood coagulation analysis of SD rats in satellite groups (6 weeks) of sub-chronic toxicity; Figure S6: Effect of CCSKO on blood biochemistry parameters of SD rats in satellite groups (6 weeks) of sub-chronic toxicity; Figure S7: Effect of CCSKO on blood electrolytes of SD rats in satellite groups (6 weeks) of sub-chronic toxicity; Figure S8: Effect of CCSKO on urinalysis of SD rats in satellite groups (6 weeks) of sub-chronic toxicity; Figure S9: Effects of CCSKO on organ weight of SD rats in satellite groups (6 weeks) of sub-chronic toxicity; Figure S10: Histological observations of SD rats in experiment groups (13 weeks) of sub-chronic toxicity in male groups (×100); Figure S11: Histological observations of SD rats in experiment groups (13 weeks) of sub-chronic toxicity in female groups (×100); Figure S12: Histological observations of SD rats in satellite groups (6 weeks) of sub-chronic toxicity in male groups (×100). Figure S13: Histological observations of SD rats in satellite groups (6 weeks) of sub-chronic toxicity in female groups (×100).
