# Supplementary material for: Edible Safety Evaluation of Cinnamomum camphora Seed Kernel Oil: Sub-Chronic Toxicity and Teratogenicity Assessments
Source: Foods. 2025 Jun 17;14(12):2116. doi: 10.3390/foods14122116 (PMC12192236; doi:10.3390/foods14122116)
Supplement: Supplementary file 1 [file foods-14-02116-s001.zip › foods-3700730-supplementary.pdf]

Supplementary information

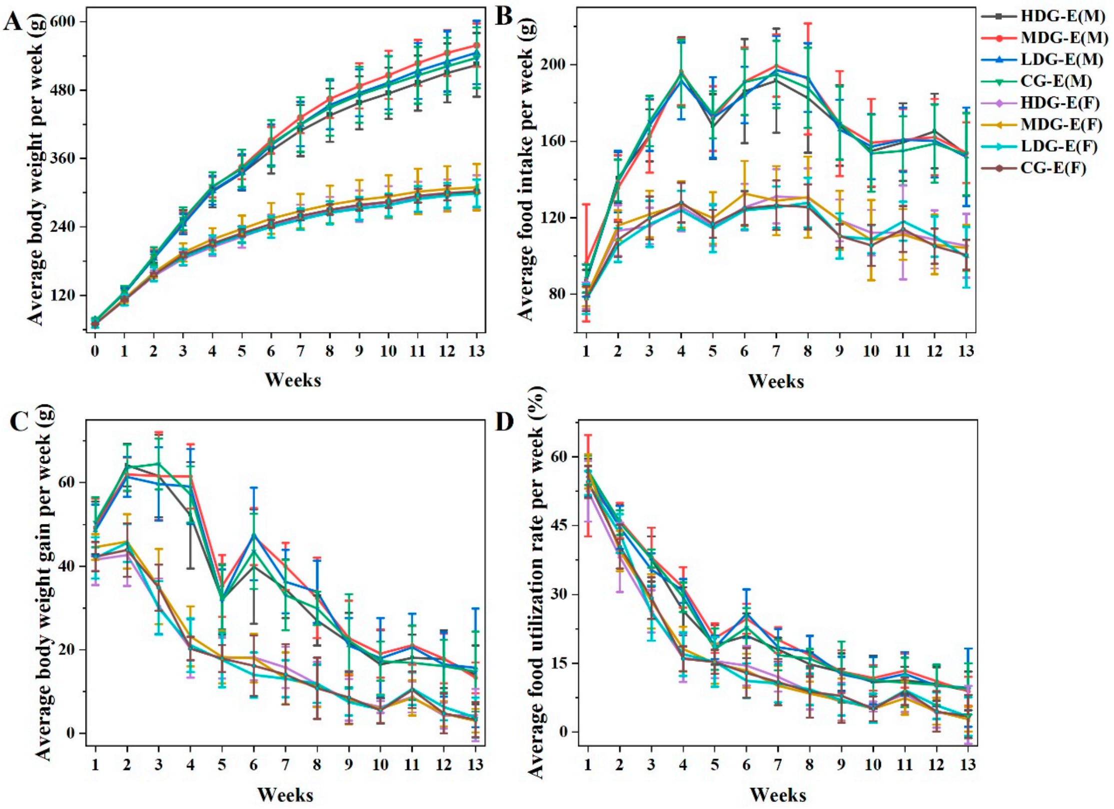

Figure S1. Effects of CCSKO on body weight (**A**), food intake (**B**), body weight gain (**C**) and food utilization rate (**D**) of SD rats in experiment groups (13 weeks) of sub-chronic toxicity. HDG-E(M), MDG-E(M), LDG-E(M), and CG-E(M) indicate high-dose group (4 mL/kg BW), medium-dose group (2 mL/kg BW), low-dose group (1 mL/kg BW), and control group (0 mL/kg BW) of male rats in experiment groups, respectively; HDG-E(F), MDG-E(F), LDG-E(F), and CG-E(F) indicate high-dose group (4 mL/kg BW), medium-dose group (2 mL/kg BW), low-dose group (1 mL/kg BW), and control group (0 mL/kg BW) of female rats in experiment groups, respectively. Data are expressed as mean ± SD (n=10).

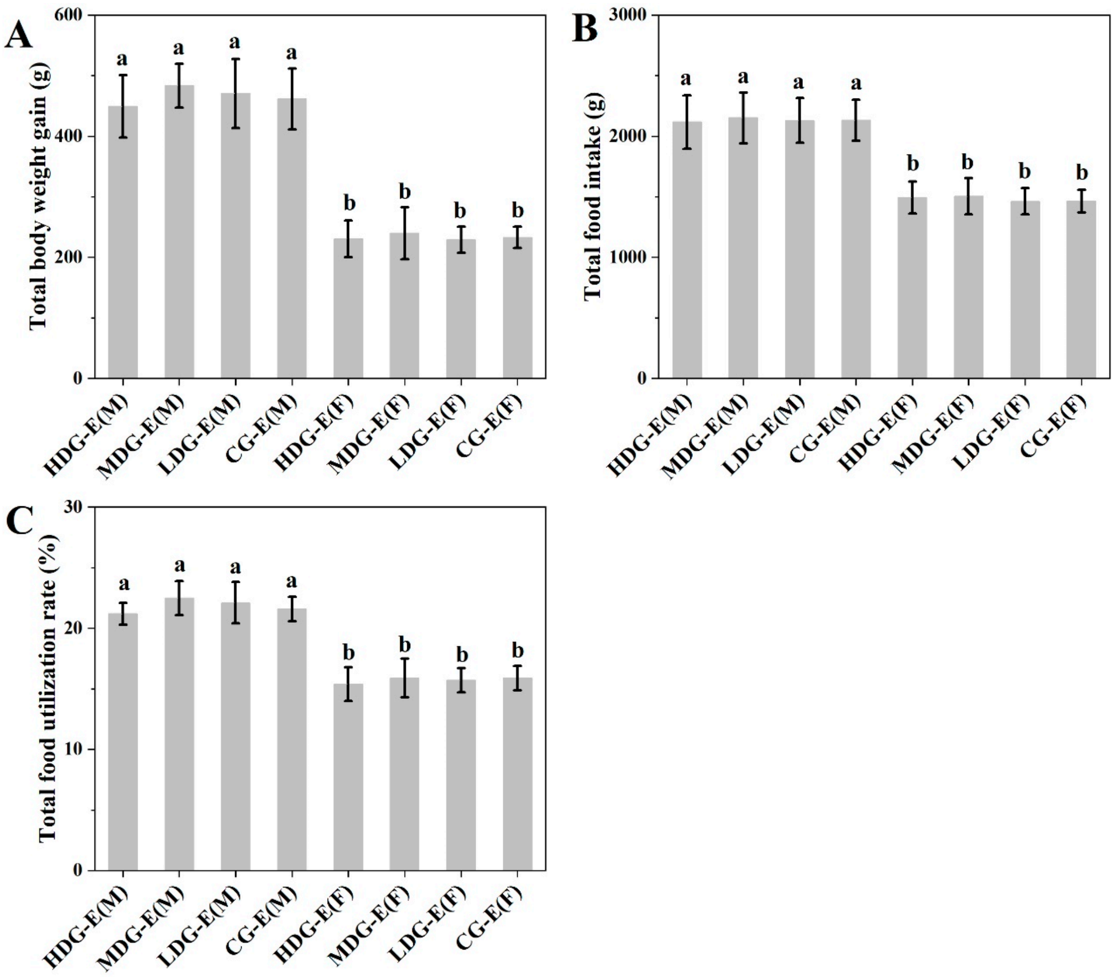

Figure S2. Effects of CCSKO on total body weight gain (**A**), total food intake (**B**) and total food utilization rate (**C**) of SD rats in experiment groups (13 weeks) of sub-chronic toxicity. HDG-E(M), MDG-E(M), LDG-E(M), and CG-E(M) indicate high-dose group (4 mL/kg BW), medium-dose group (2 mL/kg BW), low-dose group (1 mL/kg BW), and control group (0 mL/kg BW) of male rats in experiment groups, respectively; HDG-E(F), MDG-E(F), LDG-E(F), and CG-E(F) indicate high-dose group (4 mL/kg BW), medium-dose group (2 mL/kg BW), low-dose group (1 mL/kg BW), and control group (0 mL/kg BW) of female rats in experiment groups, respectively. Data are expressed as mean ± SD (n=10). No significant differences were found between control and treatment groups.

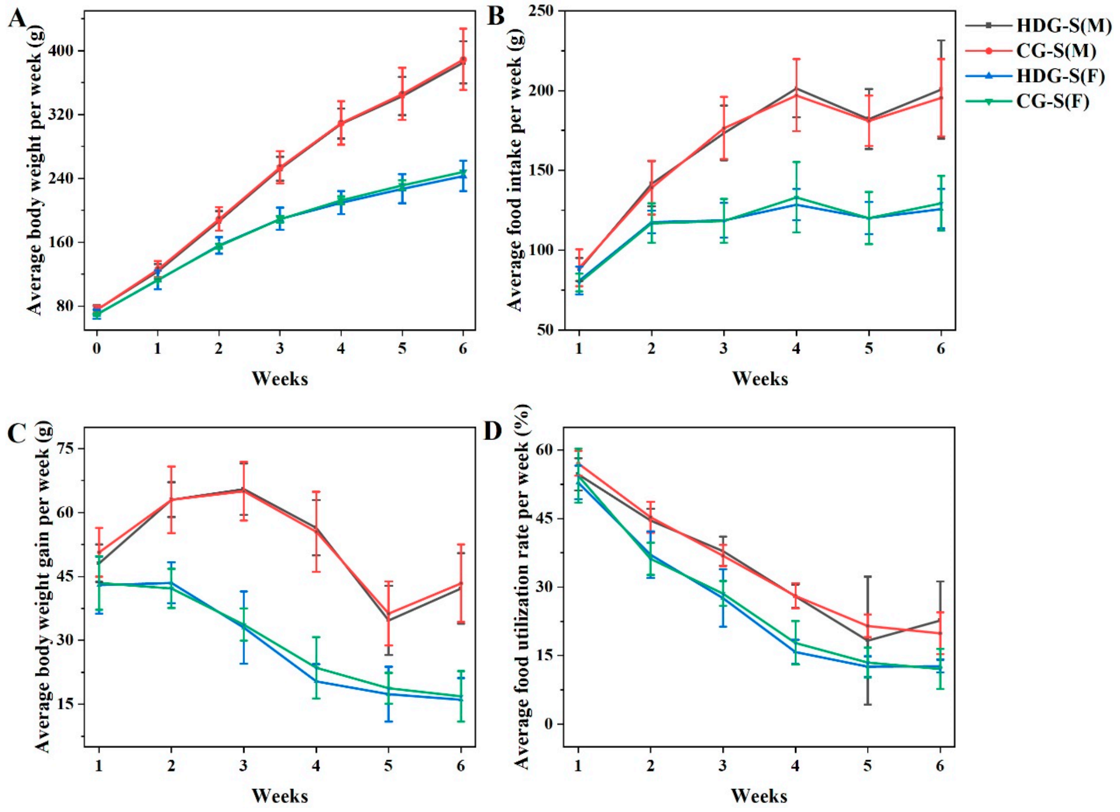

Figure S3. Effects of CCSKO on body weight (**A**), food intake (**B**), body weight gain (**C**) and food utilization rate (**D**) of SD rats in satellite groups (6 weeks) of sub-chronic toxicity. HDG-S(M) and CG-S(M) indicate high-dose group (4 mL/kg BW) and control group (0 mL/kg BW) of male rats in satellite groups, respectively; HDG-S(F) and CG-S(F) indicate high-dose group (4 mL/kg BW) and control group (0 mL/kg BW) of female rats in satellite groups, respectively. Data are expressed as mean ± SD (n=10).

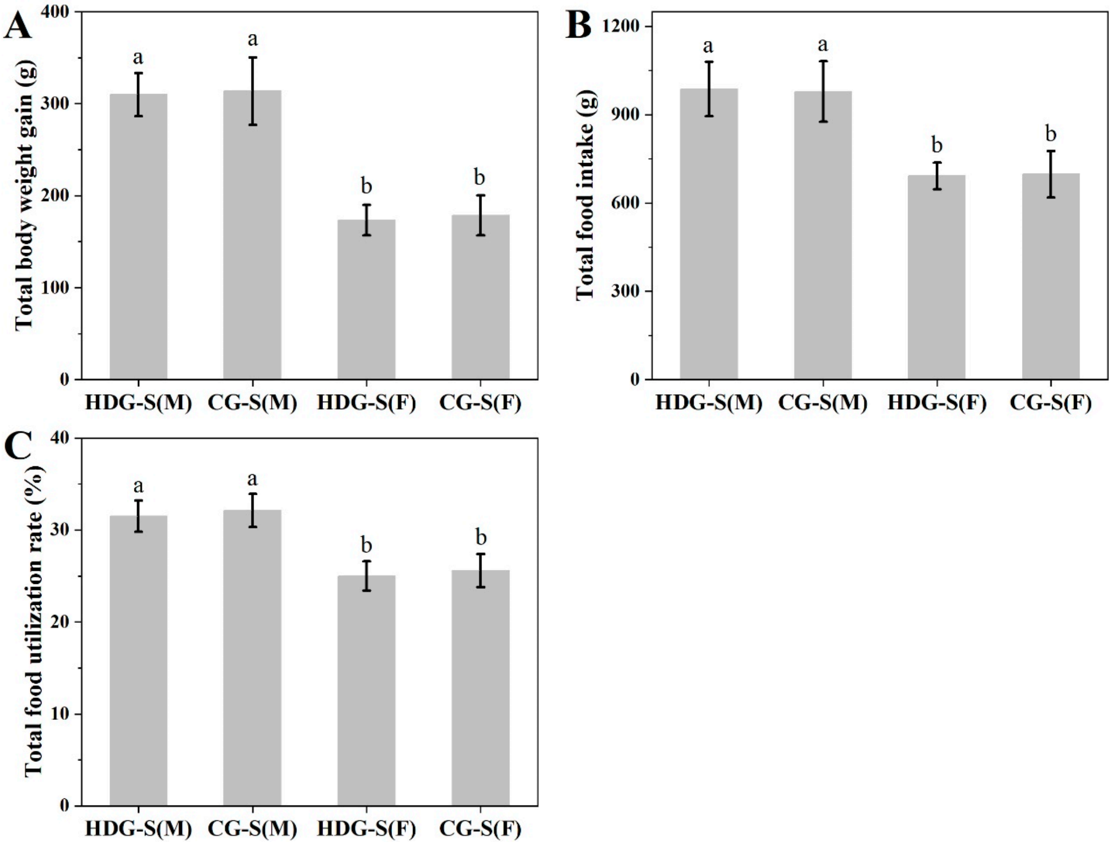

Figure S4. Effects of CCSKO on total body weight gain (**A**), total food intake (**B**) and total food utilization rate (**C**) of SD rats in satellite groups (6 weeks) of sub-chronic toxicity. HDG-S(M) and CG-S(M) indicate high-dose group (4 mL/kg BW) and control group (0 mL/kg BW) of male rats in satellite groups, respectively; HDG-S(F) and CG-S(F) indicate high-dose group (4 mL/kg BW) and control group (0 mL/kg BW) of female rats in satellite groups, respectively. Data are expressed as mean ± SD (n=10). No significant differences were found between control and treatment groups.

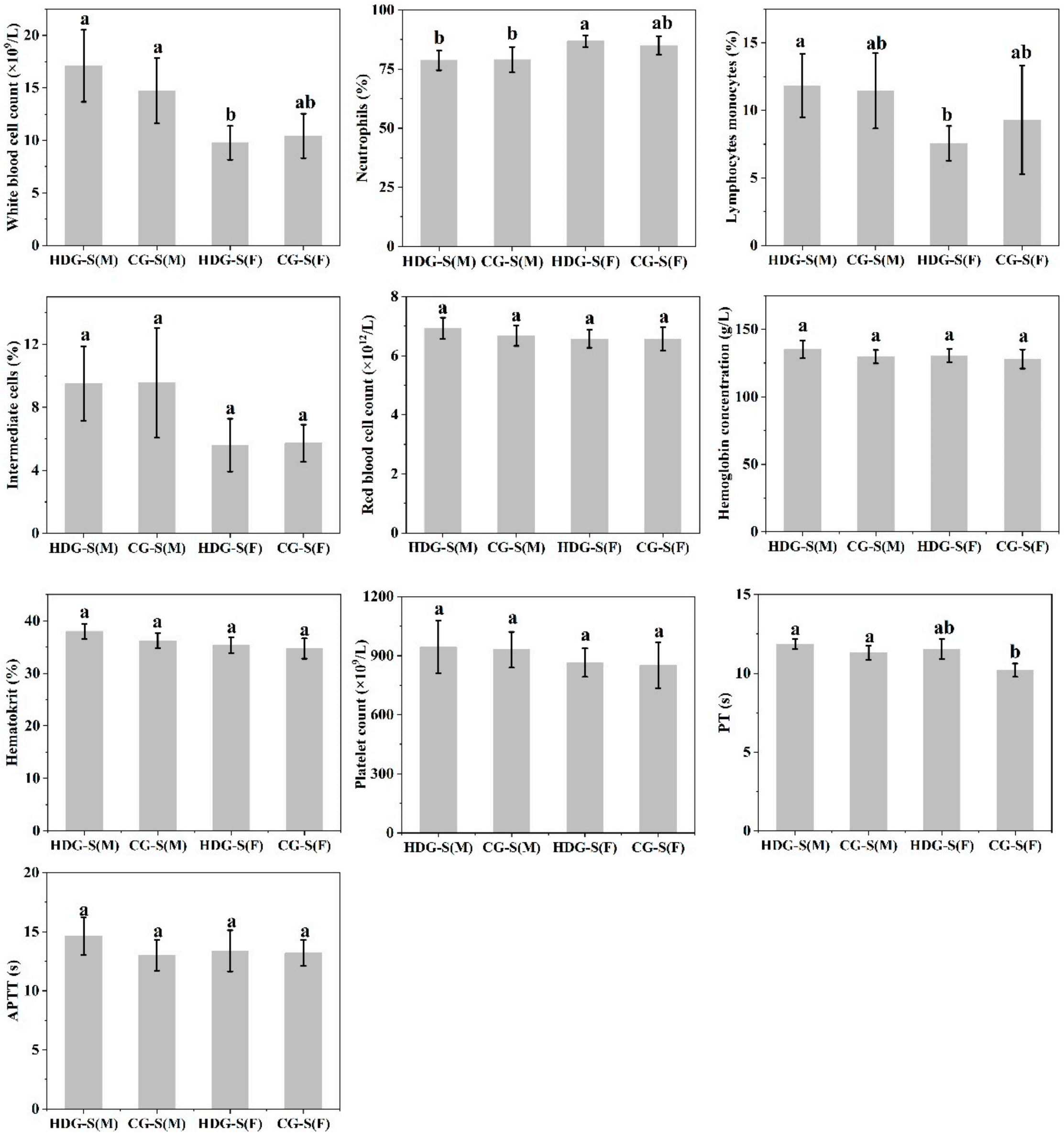

Figure S5. Effect of CCSKO on blood routine, leukocyte differential count and blood coagulation analysis of SD rats in satellite groups (6 weeks) of sub-chronic toxicity. HDG-S(M) and CG-S(M) indicate high-dose group (4 mL/kg BW) and control group (0 mL/kg BW) of male rats in satellite groups, respectively; HDG-S(F) and CG-S(F) indicate high-dose group (4 mL/kg BW) and control group (0 mL/kg BW) of female rats in satellite groups, respectively. PT, prothrombin time; APTT, activated partial thromboplastin time. Data are expressed as mean ± SD (n=10). No significant differences were found between control and treatment groups.

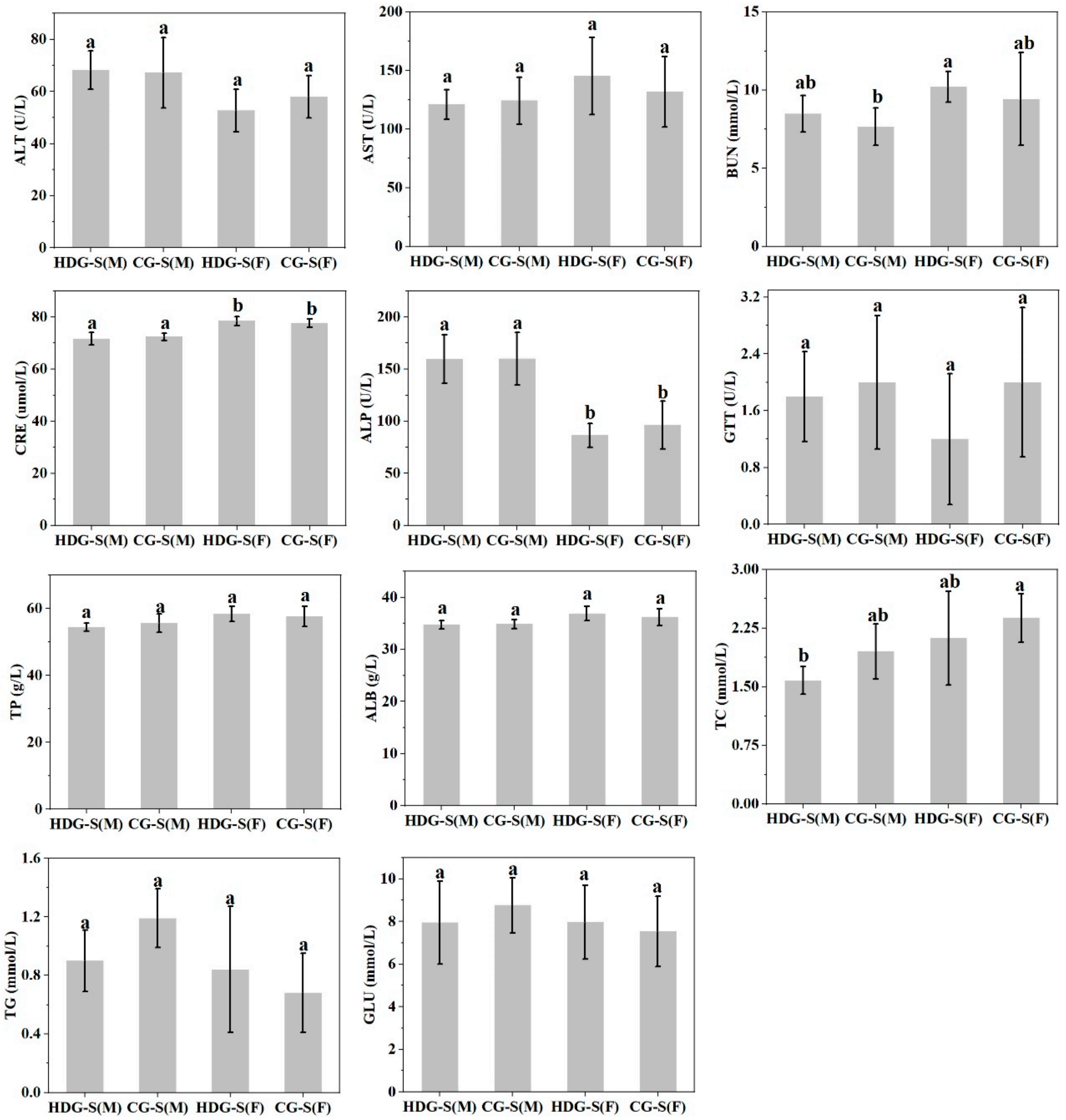

Figure S6. Effect of CCSKO on blood biochemistry parameters of SD rats in satellite groups (6 weeks) of sub-chronic toxicity. HDG-S(M) and CG-S(M) indicate high-dose group (4 mL/kg BW) and control group (0 mL/kg BW) of male rats in satellite groups, respectively; HDG-S(F) and CG-S(F) indicate high-dose group (4 mL/kg BW) and control group (0 mL/kg BW) of female rats in satellite groups, respectively. ALT, alanine aminotransferase; AST, aspartate aminotransferase; BUN, blood urea nitrogen; CRE, creatinine; ALP, alkaline phosphatase; GTT, glutamyl transpeptidase; TP, total protein; ALB, albumin; TC, total cholesterol; TG, triglycerides; GLU, glucose. Data are expressed as mean ± SD (n=10). No significant differences were found between control and treatment groups.

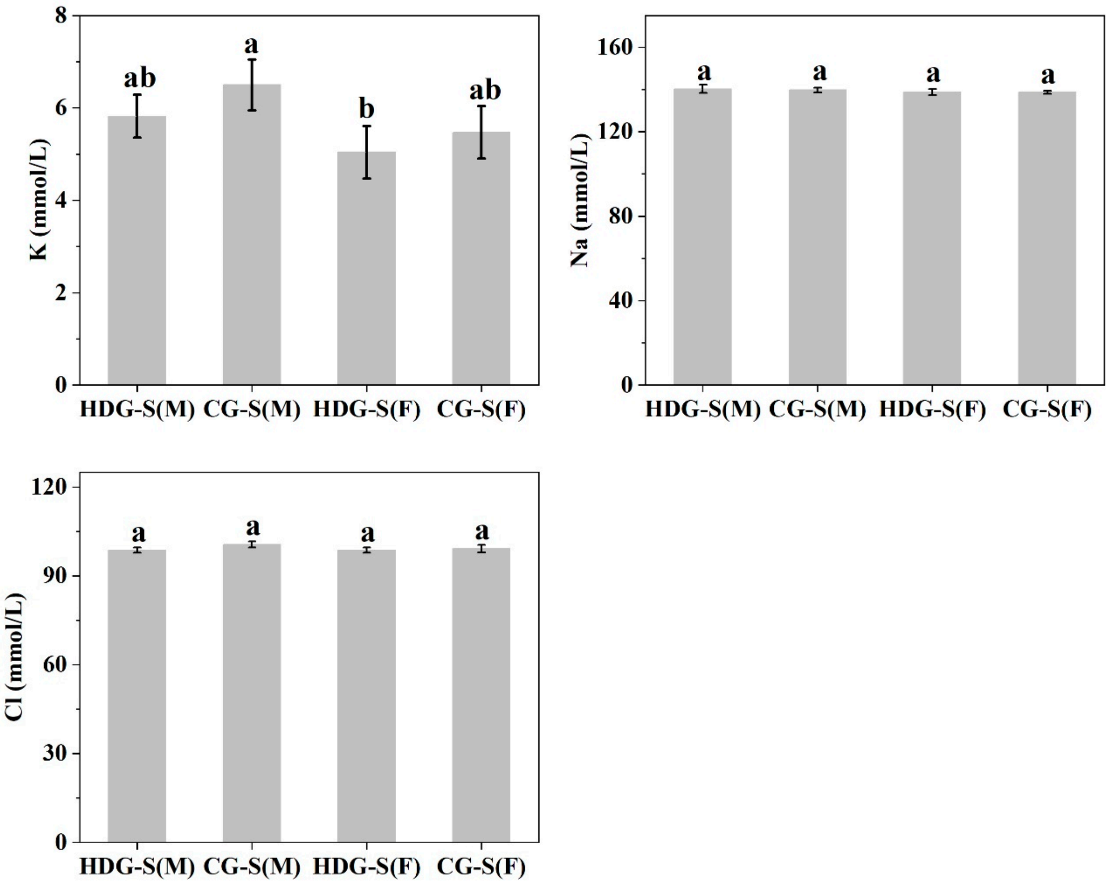

Figure S7. Effect of CCSKO on blood electrolytes of SD rats in satellite groups (6 weeks) of sub-chronic toxicity. HDG-S(M) and CG-S(M) indicate high-dose group (4 mL/kg BW) and control group (0 mL/kg BW) of male rats in satellite groups, respectively; HDG-S(F) and CG-S(F) indicate high-dose group (4 mL/kg BW) and control group (0 mL/kg BW) of female rats in satellite groups, respectively. Data are expressed as mean ± SD (n=10). No significant differences were found between control and treatment groups.

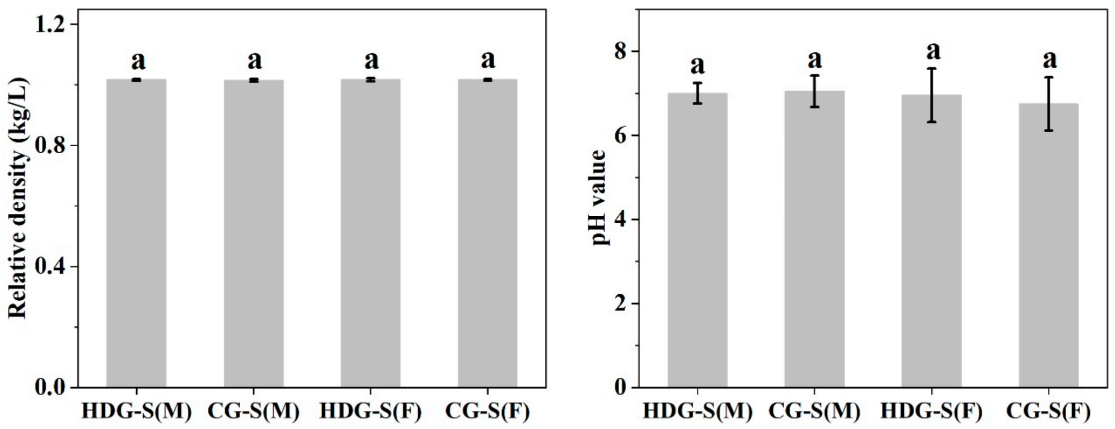

Figure S8. Effect of CCSKO on urinalysis of SD rats in satellite groups (6 weeks) of sub-chronic toxicity. HDG-S(M) and CG-S(M) indicate high-dose group (4 mL/kg BW) and control group (0 mL/kg BW) of male rats in satellite groups, respectively; HDG-S(F) and CG-S(F) indicate high-dose group (4 mL/kg BW) and control group (0 mL/kg BW) of female rats in satellite groups, respectively. Data are expressed as mean ± SD (n=10). No significant differences were found between control and treatment groups.

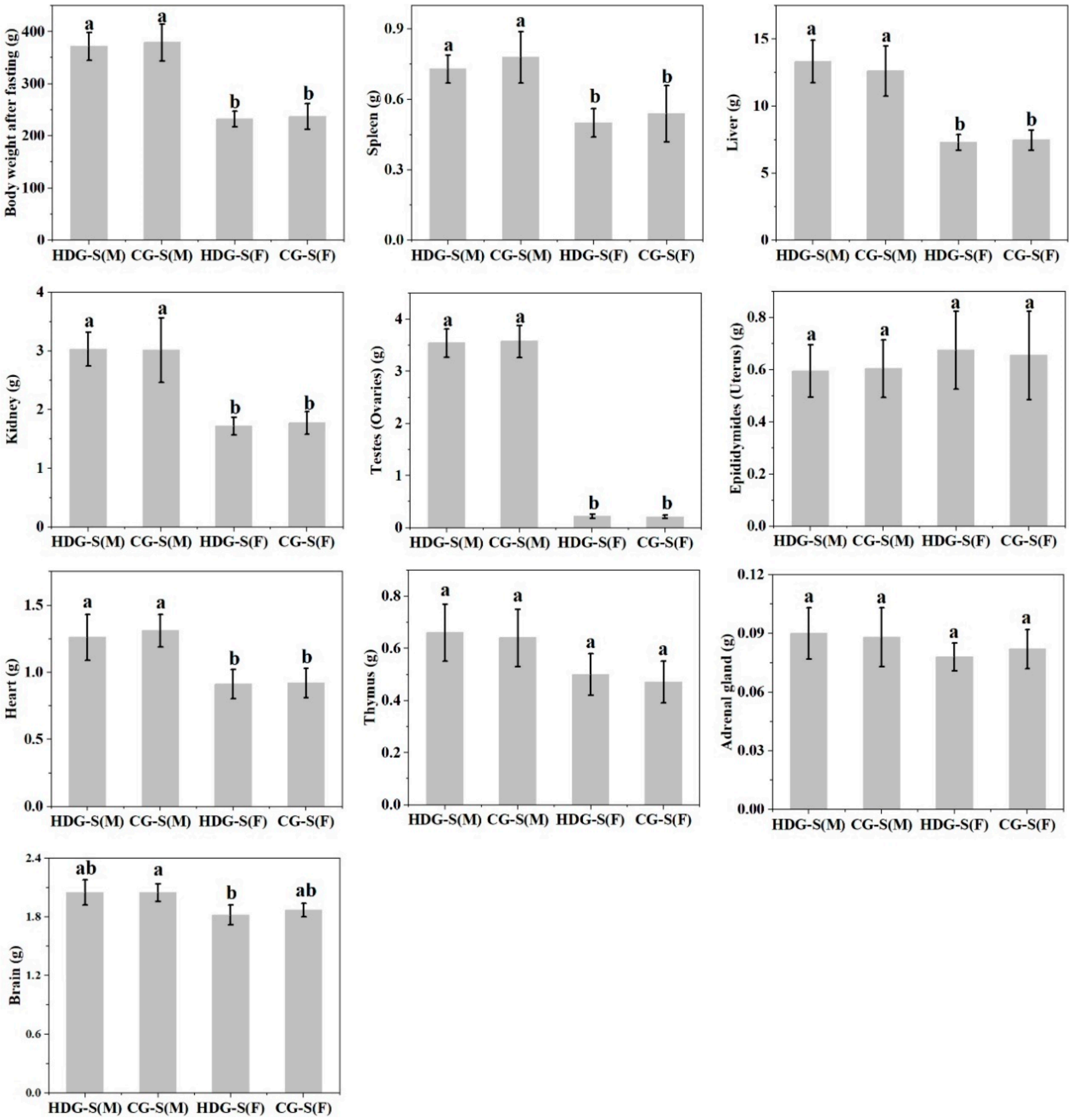

Figure S9. Effects of CCSKO on organ weight of SD rats in satellite groups (6 weeks) of sub-chronic toxicity. HDG-S(M) and CG-S(M) indicate high-dose group (4 mL/kg BW) and control group (0 mL/kg BW) of male rats in satellite groups, respectively; HDG-S(F) and CG-S(F) indicate high-dose group (4 mL/kg BW) and control group (0 mL/kg BW) of female rats in satellite groups, respectively. Data are expressed as mean ± SD (n=10). No significant differences were found between control and treatment groups.

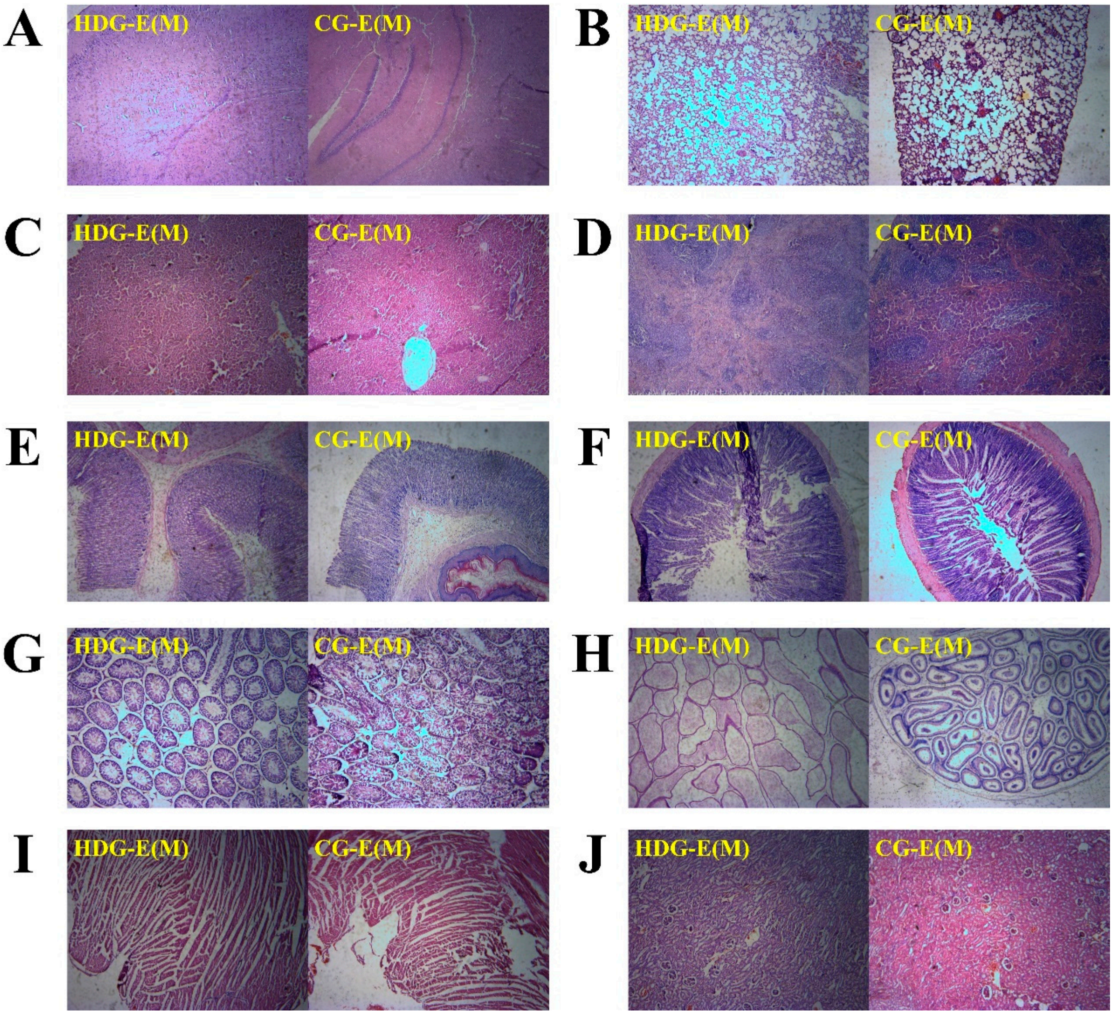

Figure S10. Histological observations of SD rats in experiment groups (13 weeks) of sub-chronic toxicity in male groups (× 100). (**A**) Brain. (**B**) Lung. (**C**) Liver. (**D**) Spleen. (**E**) Stomach. (**F**) Intestine. (**G**) Testis and ovary. (**H**) Epididymis and uterus. (**I**) Heart. (**J**) Kidneys. HDG-E(M) and CG-E(M) indicate high-dose group (4 mL/kg BW) and control group (0 mL/kg BW) of male rats in experiment groups, respectively.

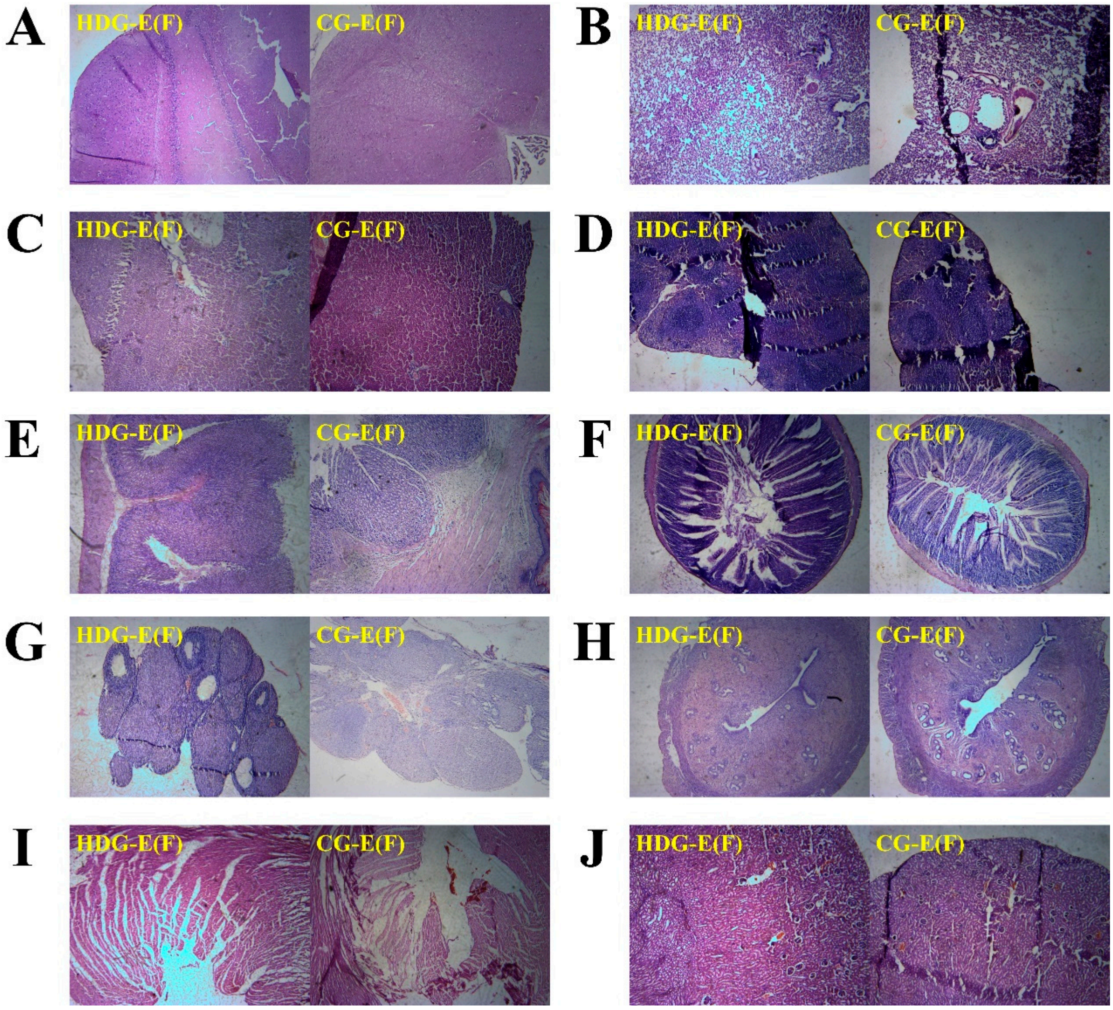

Figure S11. Histological observations of SD rats in experiment groups (13 weeks) of sub-chronic toxicity in female groups (× 100). (**A**) Brain. (**B**) Lung. (**C**) Liver. (**D**) Spleen. (**E**) Stomach. (**F**) Intestine. (**G**) Testis and ovary. (**H**) Epididymis and uterus. (**I**) Heart. (**J**) Kidneys. HDG-E(M) and CG-E(M) indicate high-dose group (4 mL/kg BW) and control group (0 mL/kg BW) of male rats in experiment groups, respectively.

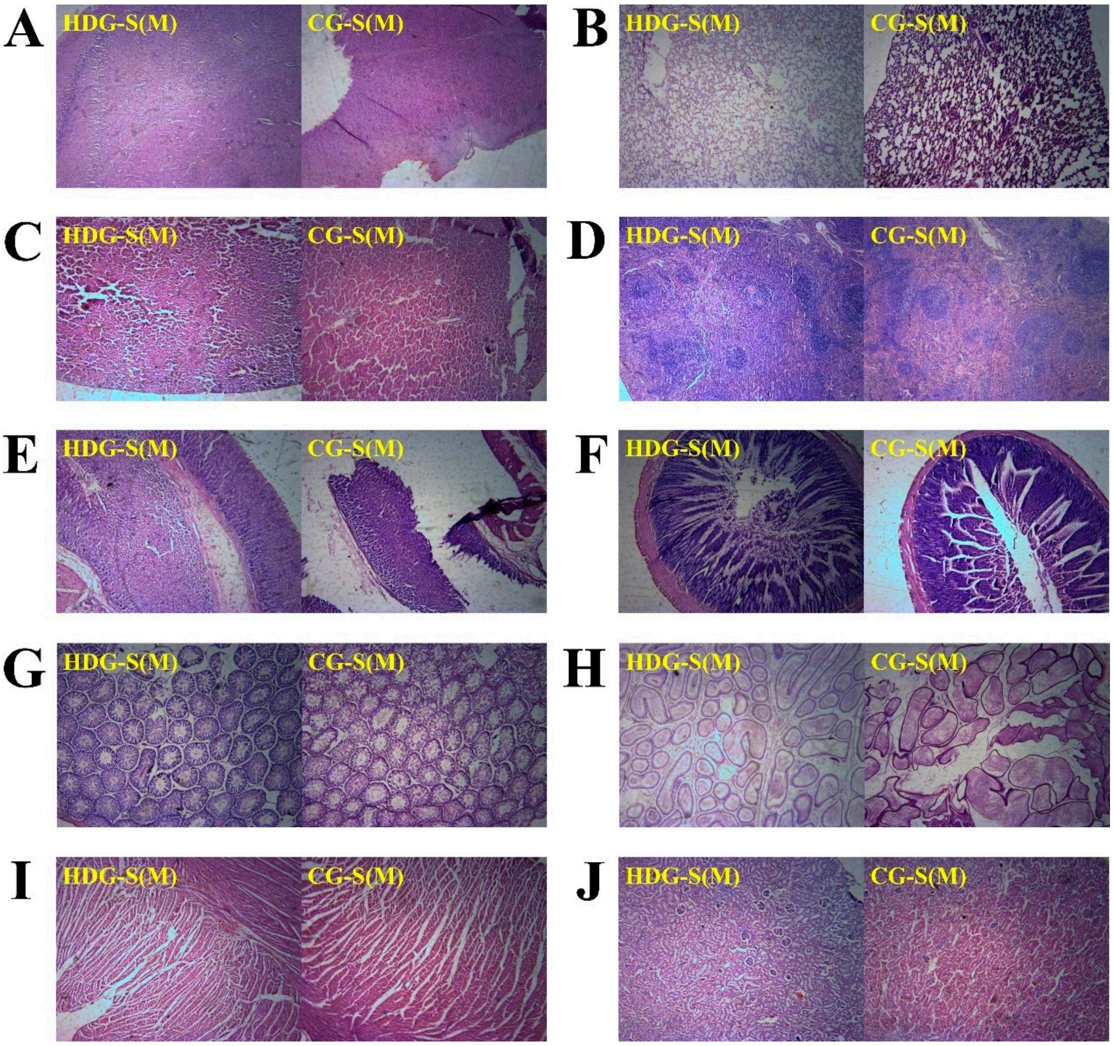

Figure S12. Histological observations of SD rats in satellite groups (6 weeks) of sub-chronic toxicity in male groups (× 100). (**A**) Brain. (**B**) Lung. (**C**) Liver. (**D**) Spleen. (**E**) Stomach. (**F**) Intestine. (**G**) Testis and ovary. (**H**) Epididymis and uterus. (**I**) Heart. (**J**) Kidneys. HDG-S(M) and CG-S(M) indicate high-dose group (4 mL/kg BW) and control group (0 mL/kg BW) of male rats in satellite groups, respectively.

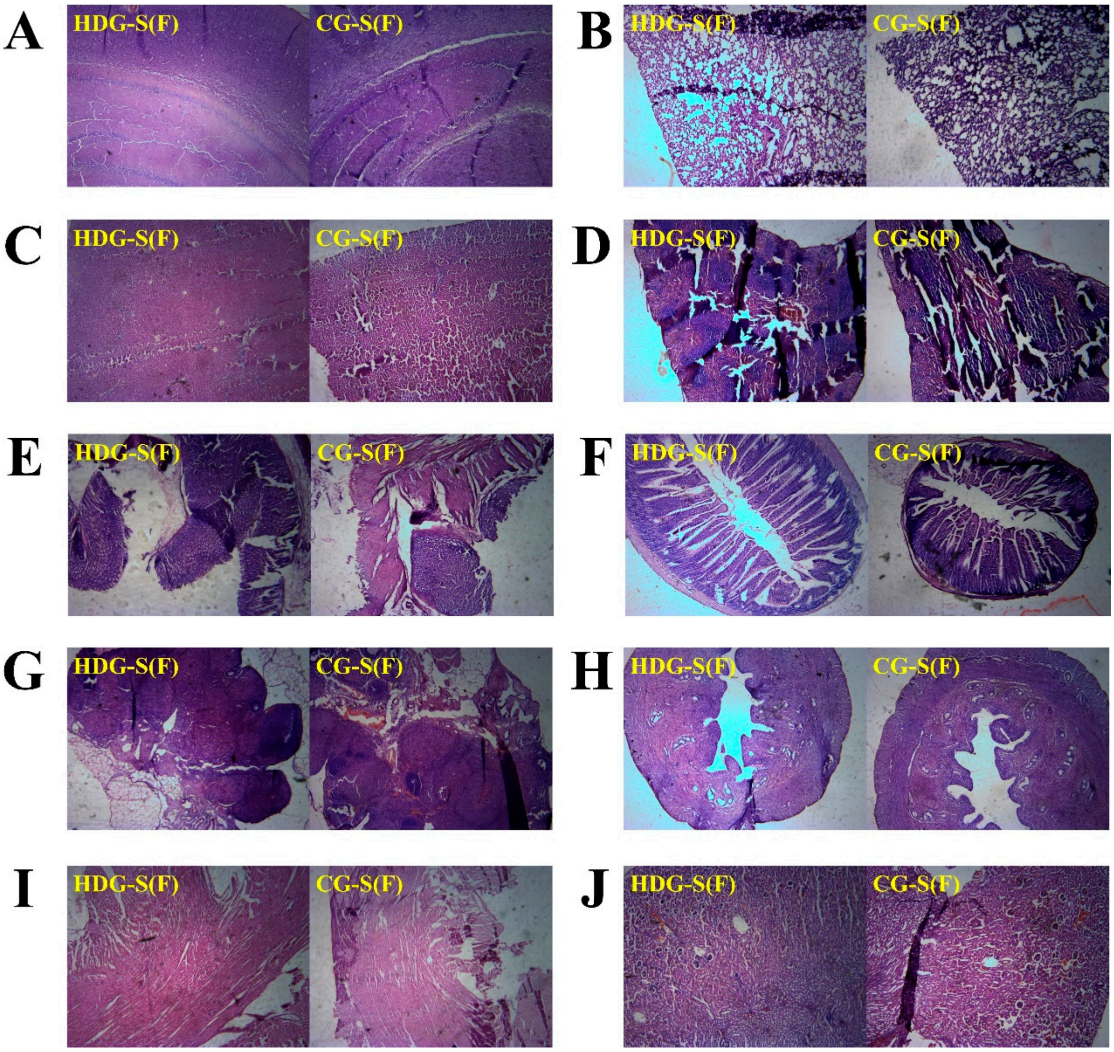

Figure S13. Histological observations of SD rats in satellite groups (6 weeks) of sub-chronic toxicity in female groups (× 100). (**A**) Brain. (**B**) Lung. (**C**) Liver. (**D**) Spleen. (**E**) Stomach. (**F**) Intestine. (**G**) Testis and ovary. (**H**) Epididymis and uterus. (**I**) Heart. (**J**) Kidneys. HDG-S(F) and CG-S(F) indicate high-dose group (4 mL/kg BW) and control group (0 mL/kg BW) of female rats in satellite groups, respectively.
